# Transformer mineral oil ingestion induces systemic sub-acute toxicity in Wistar rats

**DOI:** 10.1016/j.heliyon.2019.e02998

**Published:** 2019-12-08

**Authors:** Grace N. Otunga, Geoffrey K. Maiyoh, Benson N. Macharia, Vivian C. Tuei

**Affiliations:** a Department of Chemistry and Biochemistry, School of Science, University of Eldoret, P.O Box 1125-30100, Eldoret, Kenya; b Department of Medical Biochemistry, School of Medicine, Moi University, P.O Box 4606-30100, Eldoret, Kenya; c Department of Human Pathology and Forensic Medicine, School of Medicine, Moi University, P.O Box 4606-30100, Eldoret, Kenya

**Keywords:** Biochemistry, Toxicology, Hematological system, Pathology, Laboratory medicine, Toxicity, Transformer mineral oil, Ingestion, Rat, Sub-acute

## Abstract

We investigated the potential toxicities associated with the sub-acute ingestion of transformer mineral oil (TMO) at a heated low dose (HLD-50 mg/kg), heated high dose (HHD-500 mg/kg) and unheated high dose (UHD-500 mg/kg) in Wistar rats. There were increases in red blood cells and haemoglobin levels in HHD females and UHD males respectively versus control. The serum total proteins, albumin, and creatinine of the HHD females showed a significant increase versus control. The HHD males and UHD groups showed significant increase in liver malondialdehyde versus control. The livers of HHD groups had bile duct proliferation while those of HLD females and UHD groups showed focal areas of periportal chronic inflammation. HHD groups had kidneys with mild chronic inflammation and the HHD and UHD groups showed small intestines with chronic inflammation. In conclusion, sub-acute oral administration of TMO induced various degrees of dermal, haematological, hepatic, renal and small-intestinal toxicities in rats.

## 1 Introduction

Transformer mineral oil (TMO) is a clear liquid with an odour of petroleum. It is used as insulating oil in transformers because it is stable at high temperatures (Modenes et al., 2018). Crude oil or petroleum has always been the primary source of TMO. However, in the past few decades, siloxane-based synthetic fluids and C7-, C8-based fatty acid ester products, have also been used as sources of TMO in various parts of the world (Kaplan et al., 2010). Petroleum-based transformer fluids are normally divided into paraffinic and naphthenic-rich oils (Kaplan et al., 2010; Wang et al., 2018). TMO has a complex structure of hydrocarbon molecules and contains the following main components: paraffin (10–15 %), naphthyl or cycloparaffins (60–70 %), aromatics (15–20%), asphalt resinous substance (2.1 %), sulphur compounds (<1%), nitrogen compounds (<0.8 %), naphthenic acid (<0.02 %), and antioxidant additives/ionol (0.2–0.5%) (GlobeCore).

The Kenya Power and Lighting Company (KPLC) is the leading distributor of electricity throughout the country and consequently is a highest consumer of TMO. Several other local companies/industries in Kenya similarly use TMO in transformers and capacitors as insulating oil and a coolant. In 2015, Al Jazeera featured an article on vandalism of electrical transformers in Kenya to steal “a viscous fluid” that is later sold as cooking oil for roadside cooking stalls (Iraki, 2014). Apparently, TMO is not easily oxidized and is heat stable and so can be used repeatedly for a longer time and therefore some vendors opt to blend cooking edible oils with the TMO (Oriedo, 2010). Many Kenyans thus unknowingly ingest TMO mainly through consumption of commercial deep fried foods such as French fries, popularly known as chips, and local doughnuts called ‘mandazi’ amongst other foods prepared in a similar manner. There have been various toxicological studies on mineral oil hydrocarbons (MOH) of which MOH comprise complex mixtures, principally of straight and branched open-chain alkanes (paraffins), largely alkylated cycloalkanes (naphthenes), collectively classified as mineral oil saturated hydrocarbons and mineral oil aromatic hydrocarbons (EFSA, 2012). Earlier toxicological studies of MOHs in Fischer-344 rats showed that the higher molecular-sized hydrocarbons (microcrystalline waxes and the higher viscosity oils) were without biological effects (Baldwin et al., 1992; Smith et al., 1996). On the other hand, paraffin waxes and low- to mid-viscosity oils produced biological effects that were inversely related to molecular weight, viscosity, and melting point. In another study, findings indicated that the size and the structure of individual MOH components play a role both in determining their propensity to accumulate in different tissues and in the severity of any response that they elicit after accumulation (Scotter et al., 2003). Other comparative toxicological studies in rodents that ensued however indicated that the Fischer-344 rat might have been particularly sensitive to the lower viscosity/molecular weight MOHs (Griffis et al., 2010). Noteworthy though is that in rat, bioaccumulation of MOH can lead to formation of microgranulomas in liver (EFSA, 2012; Griffis et al., 2010; Nygaard et al., 2019). In humans exposed to MOH, lipogranulomas have been observed in liver, spleen, and lymph nodes but have not been observed to progress to significant lesions (Carlton et al., 2001; Fleming et al., 1998). In a recent comprehensive review on the role of diet in the rising incidence rates of cancer in Kenya, Maiyoh et al. found that the accidental/intentional inclusion of various chemical substances (including TMO) in Kenyan diets may play a crucial role (Maiyoh and Tuei, 2019). There are also many documented studies on exposure of fuel oils and other petroleum products to animal models for toxicity studies (Azeez et al., 2013; EFSA, 2012; Njoroge et al., 2015; Poon et al., 2004; Sunmonu and Oloyede, 2008), but altogether there are no sub-acute and chronic oral toxicity studies of TMO, despite the complex mixture of hydrocarbons in its constitution (Godinho et al., 2014). Furthermore, there are no sub-acute and chronic oral toxicity studies done on effects of using TMO in cooking and its consumption. Indeed, in their scientific opinion of 2012 on mineral oil hydrocarbons (MOH) in food, the EFSA CONTAM Panel concluded that exposure to MOH via food in Europe was of potential concern (EFSA, 2012). However, the CONTAM Panel also indicated that in view of the complexity and the lack of information on the composition of MOH mixtures and inability to resolve them into single compounds, it is not meaningful to establish health-based guidance values on the basis of studies on individual components and hence, if possible, whole-mixture studies should be used for this purpose (EFSA, 2012). There seems to be still ongoing discussions on how to approach toxicological assessment of MOHs in food (Grob, 2018). This study was therefore designed to investigate the potential dermal, haematological, hepatic, renal and small intestinal sub-acute toxic effects due to the ingestion of TMO in rats.

## 2 Materials and methods

### 2.1 Materials

Shell Diala transformer oil G (refined naphthenic non-inhibited insulating oil of density 0.89 × 10^3^ kg/m^3^ and kinematic viscosity of <25 mm^2^/s at 20 °C was obtained from Philips Medical Systems Limited, Nairobi, Kenya. The oil can be classified as medium- and low-viscosity naphthenic mineral oil as per the Joint FAO/WHO Expert Committee on Food Additives (JECFA) system (JECFA, 2002) and is in line with the specifications set for transformer and switchgear mineral insulating oils used by KPLC, Kenya (KPLC, 2014). Corn oil (Elianto) was locally purchased from Tuskys supermarket, Eldoret, Kenya. Thiobarbituric acid (TBA) was obtained from BDH Chemicals Ltd, Poole, England. 1, 1, 3, 3-Tetramethoxypropane (TMP) was obtained from Sigma Aldrich, St. Louis, Missouri, USA. All the other chemicals used were of analytical grade.

### 2.2 Ethical statement

Animal experimentation and protocols were approved by the Research Ethics Committee of University of Eastern Africa, Baraton, Kenya (Reference; REC: UEAB/7/3/2017). The sub-acute oral toxicity study was also performed according to the Organization for Economic Co-operation and Development (OECD) Test Guideline 407 for toxicity studies (OECD, 2008).

### 2.3 Study animals

Wistar albino male and female rats *(Rattus norvergicus)* aged between 6-8 weeks and weighing between 154 g to 187 g were purchased from Department of Zoology's animal facility, Chiromo Campus, University of Nairobi, Kenya. The rats were maintained at 22–25 °C with 12 h light and 12 h dark cycles, and 50–55% humidity. The rats were housed in cages with male and female rats caged separately at the Department of Biological Science's animal house, University of Eldoret. The rats were allowed to acclimatize for ten days before experimentation.

### 2.4 Animal treatment

Following acclimatization, all animals were maintained on regular rodent chow diet (Unga Farm Care East Africa Limited, Nakuru, Kenya) and had free access to drinking water throughout the study. Animal treatments were conducted according to protocol by Poon et al. (2009) with minor modifications. The rats were divided into four groups of ten rats each (5 of either sex). The corn oil control group, here referred as corn oil control (COC) group was administered with 200 μl corn oil only. TMO was dissolved in appropriate volumes of corn oil and administered to the other groups at the following doses; 50 mg/kg body weight of heated TMO here referred to as heated low dose (HLD) group, 500 mg/kg body weight of heated TMO referred to as heated high dose (HHD) group, and 500 mg/kg body weight unheated TMO referred to as unheated high dose (UHD) group. Oral administration was performed once daily at 10.00 am by oral gavage for 28 days according to OECD Test Guidelines 407 for the Testing of Chemicals on Repeated Dose 28-Day Oral Toxicity Study in Rodents (OECD, 2008). TMO was heated separately by deep frying potato slices weighing around 1000g in about 500ml of the oil at temperatures of around 180 °C (in order to mimic the local usage of the oil for preparation of deep fried foods). Repeated heating was done for 3 consecutive hours per day for five days (Morita et al., 2008) and the heated oil was stored in a sterile container until use. There was no addition of fresh TMO during the entire heating period. The dosages were maintained throughout the study by adjusting the volumes given orally according to individual animal weekly fasting body weights and density of transformer oil (0.89 × 10^3^ kg/m^3^).

### 2.5 Physical observation and determination of body weights

The rats were observed on a daily basis for changes in aggressive behaviour (burrowing and fighting) and physical appearance of the skin and fur texture. The overnight fasting body weights were determined at the beginning of the study and on weekly basis (day 0, 7, 14, 21, and 28).

### 2.6 Animal sacrifice and collection of samples

At the end of the study (day 28), animals were euthanized and blood samples collected via cardiac puncture under mild chloroform anaesthesia. Whole blood was collected in EDTA vacutainers for haematological assay while blood for serum preparation was collected in plain tubes. Serum was obtained after centrifugation at 3000 × g for 10 min and kept at −20 °C awaiting biochemical analyses. Liver, kidney, and small intestines tissues were fixed in 10% formalin for use in histological analysis**.** Liver sections for determination of malondialdehyde (MDA) levels were stored at –20 °C until analyses.

### 2.7 Haematological and biochemical analysis

A full haemogram was performed using ADVIA 2120i haematology autoanalyzer (Siemens Healthcare GmbH, Erlangen, Germany) while biochemical parameters (alanine transaminase (ALT), total proteins (TP), albumin (ALB), globulin (GLOB), urea, creatinine (CRE), and glucose) were determined in serum using COBAS INTEGRA 400 plus auto-analyzer (Roche Diagnostics, Mannheim, Germany). Levels of malondialdehyde (MDA), an end product of lipid peroxidation were determined in liver tissue homogenate as described by Alam et al. (Alam et al., 2013).

### 2.8 Histopathological analysis of liver, kidney and small intestines

Tissues from the liver, kidney and small intestines were placed in STP 120 automatic tissue processor. 5μm-thick paraffin sections were obtained using a microtome (SLEE medical model GmbH, Mainz, Germany) and stained with haematoxylin and eosin (Rai et al., 2010). Specimens were then examined for histopathological changes under a light microscope at 40X (model CX21FSI, Olympus Corporation, Tokyo, Japan) and photomicrographs taken using Redmi Note 4 model 2016102 phone from Xiaomi Communication Company Limited, Beijing, China.

### 2.9 Statistical analysis

Quantitative data was expressed as mean ± standard error of the mean (SEM). Data was analyzed by Student's t-test and one-way analysis of variance (ANOVA). Values with p < 0.05 were considered statistically significant.

## 3 Results

### 3.1 Physical appearance and body weight changes of rats on treatment with TMO

All the rats in the control and treatment groups appeared alert and with normal behaviour (fighting and burrowing) during the entire period of experimentation. The fur of rats in the control group appeared normal and smooth during the entire study period while rats administered TMO developed rough and peeling fur from day 14 of treatment. There was also no mortality of the animals during the study.

**Figure 1:**
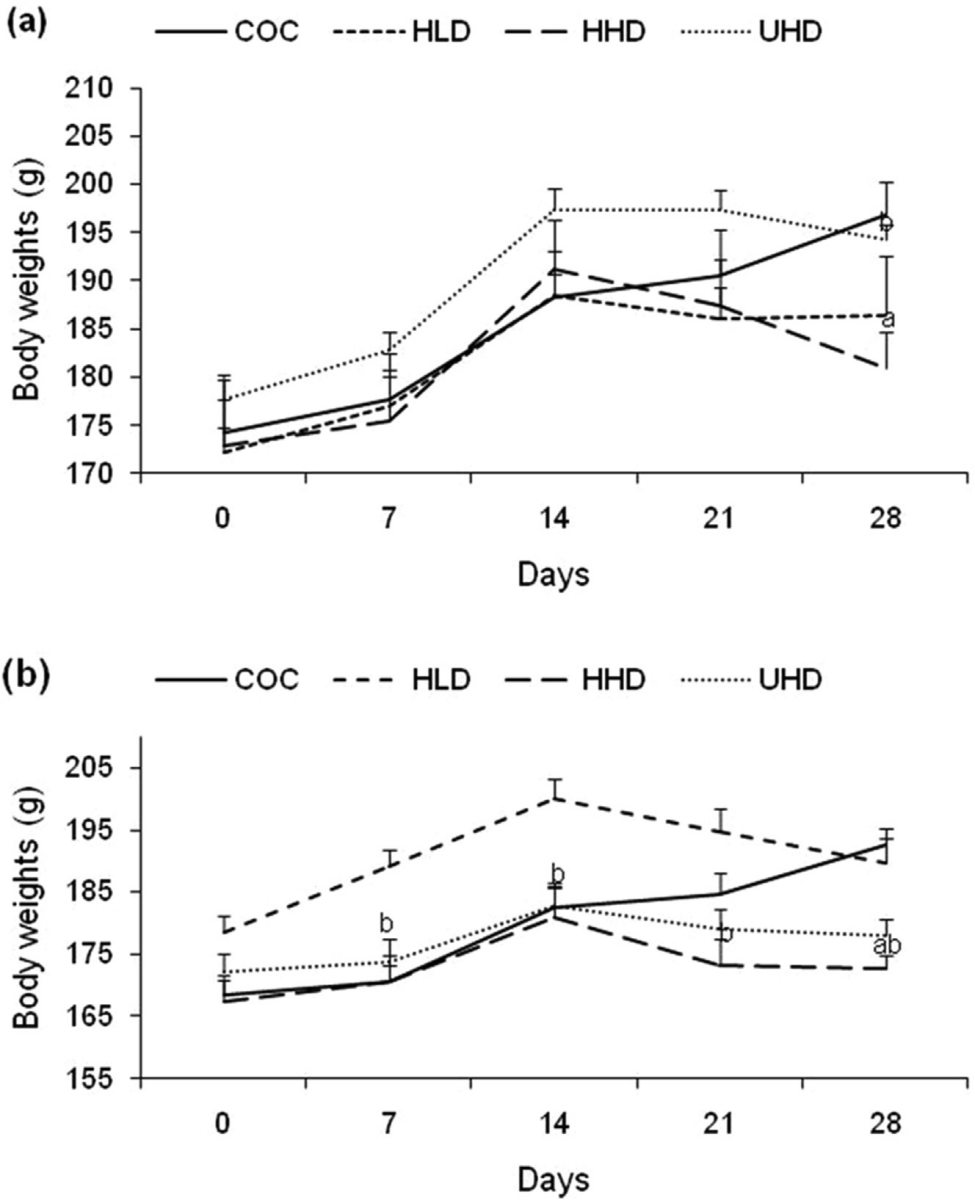
Trends in animal body weights during 28 days' oral TMO administration (Panel A: male rats and panel B: female rats). Values represent means ± SEM of five animals per group. TMO: transformer mineral oil, COC: corn oil control, HLD: heated low dose (50 mg/kg bwt), HHD: heated high dose (500 mg/kg bwt), UHD: unheated high dose (500 mg/kg bwt). ‘^a^’ significantly different from COC at p < 0.05; ‘^b^’ UHD significantly different from HHD at p < 0.05.

Male rats in both the control and TMO treated groups showed steady increase in their body weights from day 0–14 (Figure 1a) but did not reach statistical significance. Interestingly, there was little decrease in body weights as the animals progressed to day 21 followed by rapid decrease in weights to day 28 in animals treated with TMO (Figure 1a). Analysis using t-test indicated significant decrease (3%) in body weights at day 28 of HHD rats as compared to the control (Figure 1a). The UHD animals also showed a significant increase (7%) in weight at day 28 as compared to the HHD animals (Figure 1a). Male rats in the control group showed increase in body weight throughout the study period (day 0 to day 28). ANOVA results indicated that treatments administered did not significantly affect the overall body weight of male rats in treatment groups as compared to the controls and within treatment groups during the treatment period.

Like among their male counterparts, female rats in the control group had increased body weight throughout the entire period (Figure 1b). Rats treated with TMO showed body weight gain from day 0 to day 14, followed by decrease in weight from day 14 to day 28 (Figure 1b). There was a significant decrease (12%) in body weight at day 28 of animals in HHD group as compared to the control (Figure 1b). The HHD animals also showed a significant decrease in weights at day 7 (10%), 14 (9.5%), 21 (11%), and 28 (9%) as compared to the HLD group (Figure 1b). ANOVA analysis indicates that the treatment period and treatments did not significantly affect the overall body weight of female rats in treatment groups compared to the control.

### 3.2 TMO ingestion induces changes in blood cell counts in rats

When compared to the control, the male rats showed a significant decrease in red blood cells (RBC) among HLD group, significant increase in platelets (PLT) among HHD animals, and significant increase in haemoglobin (HGB) and PLT among UHD animals (Table 1). In female rats, HHD group showed significant increase in RBC and decrease in RDW compared to the control (Table 1). The UHD females showed significant decrease in red cell distribution width (RDW) as compared to the control (Table 1). Between treatment groups, the HHD males showed a significant decrease in white blood cells (WBC) and increase in RBC and HGB as compared to HLD males while the UHD females showed a significant decrease in RBC as compared to the HHD animals.

**Table 1:** Changes in haematological parameters in rats following 28 days oral administration of TMO.

Haematology parameters	Treatment groups				
	*Sex*	COC	HLD	HHD	UHD
WBC (×10^3^/μL)	M	5.78 ± 1.30	6.03 ± 0.59	3.74 ± 0.68^b^	7.02 ± 2.55
	F	4.24 ± 0.37	4.72 ± 1.16	5.70 ± 0.82	5.15 ± 0.33
RBC (×10^6^/μL)	M	8.20 ± 0.24	7.34 ± 0.23^a^	8.15 ± 0.07^b^	8.37 ± 0.10
	F	7.73 ± 0.23	8.20 ± 0.80	8.52 ± 0.17^a^	7.33 ± 0.42^c^
HGB (g/dL)	M	14.88 ± 0.23	14.4 ± 0.36	15.3 ± 0.14^b^	15.86 ± 0.22^a^
	F	14.74 ± 0.38	14.4 ± 1.04	15.6 ± 0.46	14.45 ± 0.46
HCT (%)	M	48.84 ± 2.57	45.75 ± 1.08	47.86 ± 0.78	49.66 ± 0.79
	F	45.8 ± 1.07	50.38 ± 3.31	49.45 ± 1.44	44.43 ± 1.96
MCV (fL)	M	59.5 ± 2.44	62.48 ± 1.49	58.72 ± 0.84	59.36 ± 0.83
	F	59.26 ± 0.49	65.42 ± 3.36	58.03 ± 0.59	60.78 ± 1.11
MCH (pg)	M	18.16 ± 0.42	19.68 ± 0.63	18.8 ± 0.30	18.96 ± 0.25
	F	19.06 ± 0.25	18.58 ± 0.41	18.3 ± 0.2	19.83 ± 0.60
MCHC (g/dL)	M	30.72 ± 1.42	31.48 ± 0.38	31.98 ± 0.45	31.96 ± 0.10
	F	32.18 ± 0.30	28.68 ± 1.63	31.58 ± 0.17	32.55 ± 0.43
RDW (%)	M	14.06 ± 0.23	14.88 ± 0.62	13.64 ± 0.25	14.12 ± 0.27
	F	13.94 ± 0.28	14.48 ± 0.68	12.9 ± 0.30^a^	12.9 ± 0.27^a^
PLT (x10^3^/μL)	M	547.6 ± 30.79	582.0 ± 43.45	706.4 ± 50.92^a^	787.8 ± 72.06^a^
	F	646.8 ± 75.05	508.0 ± 77.67	710.8 ± 33.57	656.25 ± 55.03

The values represent means ± SEM of five animals per group. M: male, F: female, TMO: transformer mineral oil, COC: corn oil control, HLD: heated low dose (50 mg/kg bwt), HHD: heated high dose (500 mg/kg bwt), UHD: unheated high dose (500 mg/kg bwt), WBC: white blood cells, RBC: red blood cells, HGB: haemoglobin, HCT: haematocrit, MCV: mean corpuscular volume, MCH: mean corpuscular haemoglobin, MCHC: mean corpuscular haemoglobin concentration, RDW: red cell distribution width, PLT: platelets. ‘^a^’significantly different from COC control at p < 0.05. ‘^b^’significantly different from HLD at p < 0.05. ‘^c^’significantly different from HHD at p < 0.05.

### 3.3 TMO ingestion results in alterations in hepatic and renal function biochemical indices in rat

The effect of oral TMO ingestion on both male and female rats liver function was determined by measuring serum ALT, TP, ALB, GLOB, fasting glucose as well as MDA levels in liver homogenate at the end of the treatment period. Among the male treatment groups, except for HLD, a significant decrease in ALT was observed as compared to the COC control (Table 2). The HHD males also showed a significant decline in ALT as compared to HLD animals. Except for female HHD rats that showed a significant increase in TP and ALB relative to COC controls, there were no significant alterations in TP, ALB and GLOB levels among other treatment groups (Table 2). There were no significant changes in fasting blood glucose (FBS) levels between the treatments and the control groups among the male rats (Table 2). However, the female rats showed a significant increase in glucose levels among the HLD rats (36%), HHD rats (43%), and UHD rats (57%) as compared to the COC control group (Table 2). Both the male and female rats showed no significant differences between treatment groups. Significant changes in MDA levels were observed in liver tissues of HHD males (22% increase) and UHD males (24% increase) when compared to the control (Table 2). The female UHD animals showed significant increase (34%) in MDA levels as compared to the control (Table 2). No significant changes in MDA levels were observed in HHD and UHD male and female animals as compared to HLD and HHD groups respectively (Table 2).

**Table 2:** Biochemical parameters of rats following 28 days oral administration of TMO.

Biochemical Parameters	Treatment Groups				
	*Sex*	COC	HLD	HHD	UHD
ALT (U/L)	M	81.02 ± 7.51	80.84 ± 9.78	45.3 ± 4.32^ab^	53.7 ± 4.16^a^
	F	53.12 ± 3.40	54.32 ± 5.90	48.12 ± 5.38	46.76 ± 4.92
Total protein (g/L)	M	62.52 ± 1.60	61.72 ± 1.80	58.16 ± 3.33	62.56 ± 1.80
	F	62.64 ± 0.84	66.82 ± 2.32	68.76 ± 0.96^a^	63.88 ± 3.61
Albumin (g/L)	M	37.88 ± 0.76	38.28 ± 0.83	36.41 ± 1.90	39.52 ± 0.22
	F	36.84 ± 1.61	39.78 ± 1.40	42.38 ± 1.64^a^	38.61 ± 1.86
Globulin (g/L)	M	24.62 ± 1.16	23.46 ± 1.93	22.36 ± 2.63	23.04 ± 1.66
	F	25.78 ± 1.92	27.04 ± 2.12	26.4 ± 0.90	25.28 ± 3.20
Urea (mmol/L)	M	5.92 ± 0.73	8.28 ± 1.10	7.81 ± 0.75	7.71 ± 0.45
	F	8.20 ± 0.51	7.10 ± 0.31	8.37 ± 1.06	6.34 ± 0.32^a^
Creatinine (μmol/L)	M	25.6 ± 1.44	25.0 ± 1.76	27.4 ± 1.17	26.2 ± 0.86
	F	22.4 ± 2.14	23.0 ± 0.63	29.0 ± 2.07^b^	23.2 ± 1.56
Fasting Glucose (mmol/L)	M	6.82 ± 1.24	9.15 ± 1.10	6.63 ± 1.61	6.42 ± 0.56
	F	4.88 ± 0.32	6.62 ± 0.39^a^	6.97 ± 0.49^a^	7.67 ± 0.45^a^
Liver MDA (nM)	M	772 ± 41	864 ± 44	948 ± 36^a^	956 ± 39^a^
	F	620 ± 28	672 ± 32	748 ± 87	832 ± 84^a^

The values represent means ± SEM of five animals per group. M: male, F: female, TMO: transformer mineral oil, COC: corn oil control, HLD: heated low dose (50 mg/kg bwt), HHD-TMO: heated high dose (500 mg/kg bwt), UHD: unheated high dose (500 mg/kg bwt), ALT: alanine transaminase, MDA: malondialdehyde. ‘^a^’significantly different from COC control at p < 0.05. ‘^b^’significantly different from HLD at p < 0.05.

The effect of oral TMO ingestion on both male and female rats kidney function was determined by measuring serum creatinine and urea levels at the end of the treatment period. Among the male rats, no significant differences were observed among the various TMO treated groups as compared to the control (COC) and within TMO treated groups (Table 2). Among the female rats, although there were no significant changes in urea levels among the HLD animals and HHD animals when compared to the COC control group, the UHD treated female rats showed a significant decline in urea levels (22%) in comparison to the control (Table 2). Except for the HHD group that showed a significant increase in creatinine levels (26%) when compared to the HLD group, values in other groups remained the same relative to COC controls (Table 2).

### 3.4 Effect of TMO on liver, kidney and small intestinal tissue architecture of rats

**Figure 2:**
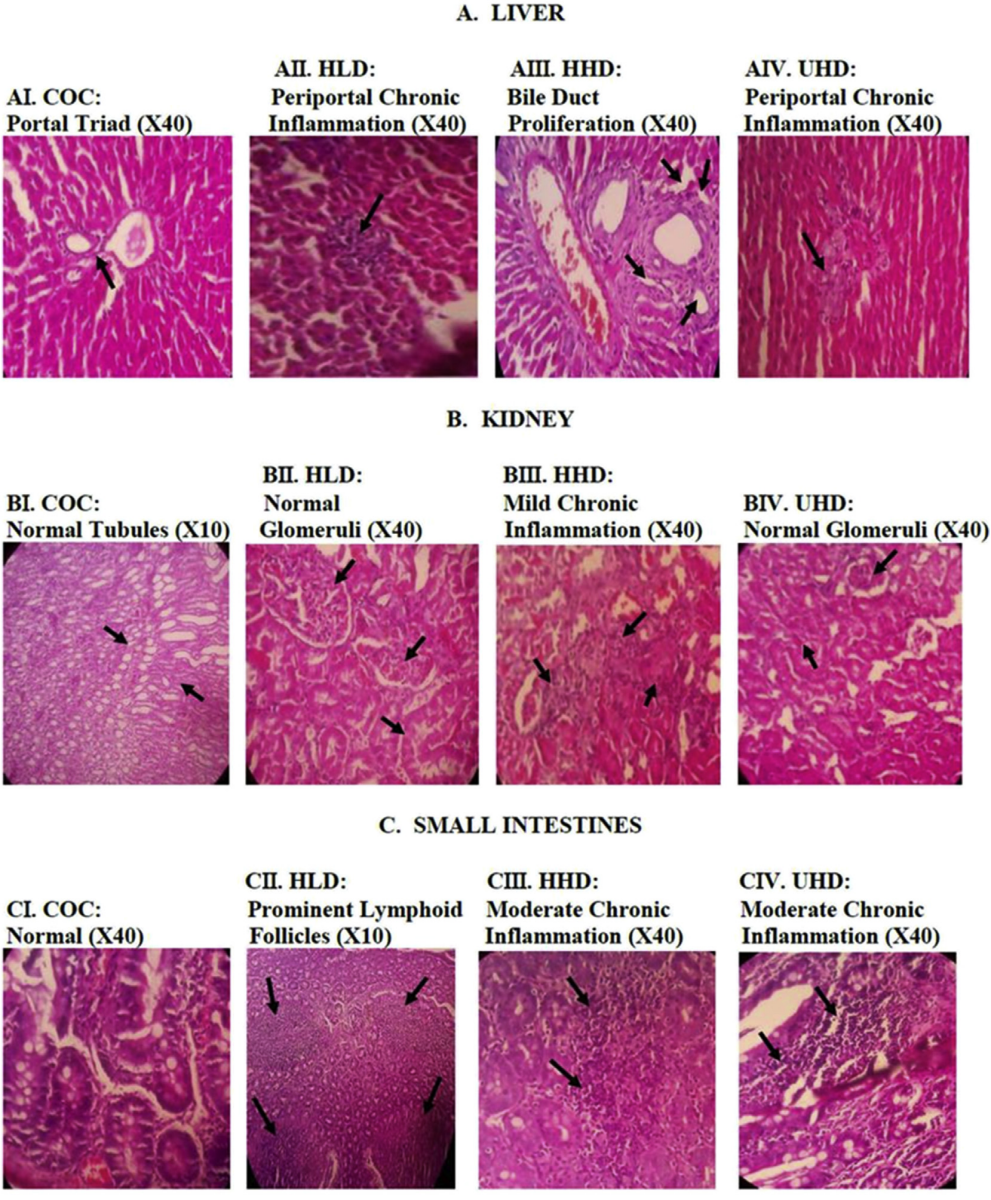
Photomicrographs of tissues of liver, kidney, and small intestines of rats treated with TMO in the sub-acute oral toxicity (Haematoxylin and eosin stained). TMO: transformer mineral oil, COC: corn oil control, HLD: heated low dose (50 mg/kg bwt), HHD: heated high dose (500 mg/kg bwt), UHD: unheated high dose (500 mg/kg bwt).

Animals in the control group showed normal liver architecture (Figure 2AI). The female HLD and UHD animals showed focal areas of mild periportal chronic inflammation (Figure 2AII and AIV respectively). The male and female animals in the HHD showed livers with bile duct proliferation (Figure 2AIII). The male and female rats in COC, HLD, and UHD groups showed normal kidneys (Figure 2BI, BII and BIV respectively). The HHD group however, showed focal areas of mild chronic inflammation (Figure 2BIII). Animals in the COC and all treated groups had small intestines tissue with lymphoid follicles (Figure 2CI–IV). Both male and female animals in HHD and UHD groups showed focal areas of chronic inflammation (Figure 2CIII and CIV respectively) which was absent in the COC group (Figure 2CI).

## 4 Discussion

Transformer mineral oil (TMO) is reportedly not easily oxidized and is heat stable and so can withstand repeated heating for a long time (Modenes et al., 2018). Due to these properties that are considered desirable, some local Kenyan fried food vendors use it to blend cooking edible oils. Consequently, many people unknowingly ingest TMO in deep fried foods such as French fries, popularly known as chips, and doughnuts (locally known as ‘mandazi’) amongst other foods. Although this information is in the public domain in Kenya (that there is vandalism of power transformers to obtain TMO for use as a blend to ordinary cooking oil/fat), to our knowledge, there are no studies that have been conducted to investigate the safety/toxicity associated with the ingestion of TMO. Towards this end, the present study sought to examine the effects of sub-acute oral ingestion of TMO to various body systems in Wistar rats. An attempt was made to mimic the manner in which TMO finds its way in human diet in Kenya. To achieve this, the TMO used for the study was initially used as a deep frying agent for potato chips (French fries) prior to its oral administration to our study animals. Results from rats ingesting heated TMO were compared against those of animals not receiving TMO (received corn oil instead) and those receiving TMO that had not been subjected to any deep frying (heating). Since, previous toxicological rodent studies have majorly focussed on exposure to mineral oil hydrocarbons (Grob, 2018), the present study particularly assessed sub-acute oral toxicity of TMO, a medium- and low-viscosity naphthenic mineral oil.

Surprisingly, there were no mortalities nor was there any significant change in animal behaviour (fighting and burrowing) during the entire study period with rats in all groups appearing alert. Whereas this may appear to be indicative of nonhazardous TMO quality, it may also represent delayed latency of some of the symptoms related to TMO ingestion. The earliest signs of toxic effects in our study were underscored by the declining body weights and the changes in animal coats that appeared at 2 weeks. The fur of rats administered with TMO became rough and started to peel off. This appears to be in agreement with earlier observations with other petroleum based products like kerosene observed to cause dermal irritation and sensitization, skin lesions and dermatitis (Maiyoh et al., 2015). This may be attributable to the presence of certain chemical substances such as the carcinogenic polycyclic aromatic hydrocarbons (PAHs) in TMO. Indeed previous studies have shown that animals exposed to PAHs contained in petroleum products exhibit substantially increased risk of skin cancer (Iyanda, 2016; Virich et al., 2008). Although potential genotoxicity/mutagenicity was not investigated in the present study, our data alongside other previous studies with petroleum based agents point to the need for future studies to determine the potential carcinogenicity of TMO in rats. Regarding the declining body weights among animals receiving TMO treatment, it was interesting to note that the onset of decline in body weights coincided with the onset of abnormalities observed in the skin. We presume that this observation could be indicative of the time about which the onset of toxic effects of TMO begun to affect not only the skin but also various body systems. The observed trends in body weights were very similar among both male and female rats.

Our investigation on the effects of TMO ingestion on blood components, showed significant decreases in red cell distribution width (RDW) of HHD and UHD groups among the female rats. RDW levels measures the variation in size of RBCs and increased values are usually indicative of the presence of cardiovascular diseases or metabolic inflammatory disorders (Felker et al., 2007; Lippi et al., 2009). Various studies have also shown a relationship between RDW and the regulation of glucose metabolism (Tripolino et al., 2016) and that RDW values could serve as predictor of glucose metabolism disturbances. Among our female rats with decreased RDW, it was interesting to note that their sugar levels were significantly increased as compared to their COC counterparts. The increased glucose levels within the treatment period remained within the published normal ranges for the species (He et al., 2017). As to why these effects on RDW and blood glucose were only observed among female rats requires further investigations. Although there was a significant increase in the PLTs levels of HHD and UHD treated male rats, the values remained within the acceptable published normal reference values for rats (He et al., 2017) and therefore may not have borne any adverse effects to the animals within the treatment period. Nonetheless, the effects of TMO treatment beyond 28 days (chronic exposure) remain to be determined and therefore future studies should be conducted either at a higher dose or for longer duration since elevated PLT is associated with clotting problems. There was a significant increase in RBCs and haemoglobin (HGB) in HHD males compared to the HLD counterparts. Like their male counterparts, the female HHD animals also showed significant increase in RBCs compared to the control group. No direct harm may arise from this effect, as on the contrary, it may actually be beneficial since RBCs increases are associated with increased capacity to carry oxygen (Njoroge et al., 2015). The UHD males also showed a significant increase in HGB compared to the control. It is interesting to note that TMO did not induce anaemic conditions as is expected with many petroleum products. The rest of the blood components including WBC, HCT, MCV and MCHC were unaltered.

Based on studies conducted on the small intestines and livers of our experimental animals, our study results also indicate that oral TMO ingestion may have toxic effects to both the hollow and the solid organs of the digestive system. For the liver, the TP and ALB levels of the HHD female animals were significantly elevated as compared to the COC animals. On the other hand, the liver of animals on TMO (both HLD and HHD, male and female) showed bile duct proliferation and chronic inflammation. Altogether, these changes may represent damages induced by TMO to the liver of these animals. A likely underlying mechanism responsible for the hepatotoxic effects could be oxidative damage attributable to the observed increase in liver MDA levels, (an index of lipid peroxidation), as observed in UHD groups. Similar findings have been observed in previous studies among animals fed on diesel (Aksoy, 2015). In rats, bioaccumulation of mineral oil saturated hydrocarbons can lead to formation of microgranulomas in liver and mesenteric lymph nodes (Nygaard et al., 2019) and particularly the microgranulomas in liver have been associated with inflammatory reactions (EFSA, 2012). In contrast, some previous studies observed significant decrease in serum ALB in rats ingesting kerosene (Njoroge et al., 2015) which were attributed to possible direct injury to hepatocytes and loss of function since plasma proteins are synthesized in the liver. It is noteworthy that our results on ALB and TP values were not corroborated by ALT values, which interestingly showed a declining trend among animals receiving TMO, and thus warrants further investigations. In respect to effects of TMO to the small intestinal architecture, the animals in the HHD and UHH groups showed focal areas of chronic inflammation in their small intestine typical of petroleum products (Njoroge et al., 2015). Chronic inflammation of the gut is usually associated with many intestinal inflammatory diseases that affect any part of the gut including the stomach, small intestines, and colon (Rubin et al., 2012) so although not investigated, the harmful effects related to TMO ingestion may thus not be restricted to the small intestines alone but may have affected other gastrointestinal (GI) structures. The damage to the small intestines was not as severe since all animals showed presence of lymphoid follicles which could be representative of normal intestinal functioning (Haley, 2017). We presume however, that prolonged exposure to TMO and/or higher doses may result to further more adverse histological changes in tissues of the GI beyond what was observed in the present sub-acute study.

As for studies on the renal system, the HHD female rats showed a significant increase in serum creatinine levels when compared to the HLD group suggesting their kidney's inability to excrete this nitrogenous waste product from the body (Azeez et al., 2013). At tissue level however, both the male and female rats in the various groups had normal kidney architecture except for HHD animals that showed focal areas of mild chronic inflammation. Chronic inflammation plays a critical role in initiation of many forms of chronic kidney diseases (Waters and Vogt, 2018). Due the presence of hydrocarbons in TMO, it can be expected that just like in other petroleum products, TMO ingestion may lead to kidney damage by free radicals generated from their metabolism (Azeez et al., 2013).

## 5 Conclusions

Overall, the mild histopathological findings from this oral sub-acute toxicity study suggest that oral sub-chronic or chronic exposures or exposures to higher doses of TMO to rats could potentially result in effects of toxicological relevance. Some of the hematological and biochemical effects reported are of questionable toxicological significance or might even be adaptive responses as there was absence of clear dose-response compounded with the limitation of low number of animals in each experimental group. Heating of TMO did not seem to lead to any additional toxicity. This study demonstrates as in other toxicological rodent studies involving short-term oral exposures to mineral oil hydrocarbons, that TMO is toxic albeit with a low order of toxicity. Future research on TMO ingestion is therefore required to further elucidate mechanisms of toxic effects observed in the present study and health effects following its chronic oral exposure.

## Declarations

### Author contribution statement

Grace N. Otunga: Conceived and designed the experiments; Performed the experiments; Analyzed and interpreted the data; Contributed reagents, materials, analysis tools or data; Wrote the paper.

Geoffrey K. Maiyoh, Vivian C. Tuei: Conceived and designed the experiments; Analyzed and interpreted the data; Wrote the paper.

Benson N. Macharia: Analyzed and interpreted the data; Contributed reagents, materials, analysis tools or data; Wrote the paper.

### Funding statement

This work was supported by the National Research Fund (NRF), Kenya (2016/2017 Masters' Research Award to Grace N. Otunga).

### Competing interest statement

The authors declare no conflict of interest.

### Additional information

No additional information is available for this paper.
